# A system dynamics analysis determining willingness to wait and pay for the implementation of data standards in clinical research

**DOI:** 10.1186/1478-4505-8-38

**Published:** 2010-12-31

**Authors:** Luciana Cofiel, Guilherme R Zammar, Amrapali J Zaveri, Jatin Y Shah, Elias Carvalho, Meredith Nahm, Gustavo Kesselring, Ricardo Pietrobon

**Affiliations:** 1 Department of Psychiatry, Institute of Psychiatry of the University of São Paulo, Dr. Ovídio Pires de Campos Street 785, 01060-970, São Paulo, Brazil; 2 Department of Medicine, Pontific Catholic University of Parana, Imaculada Conceição Street 1155, CEP 80215-90, Curitiba, Brazil; 3 Universität Leipzig, Institut für Informatik, Johannisgasse 26,D-04103 Leipzig, Germany; 4 Department of Research, Duke NUS Graduate Medical School, 8 College Road, 169857, Singapore; 5 Data Process Center, State University of Maringá, Colombo Avenue 5.790, Maringá, 87020-900, Brazil; 6 Clinical Research Informatics, Duke Translational Medicine Institute, Duke University, 2424 Erwin Road, Durham, 27705, USA; 7 Clinical Research Center, Hospital Alemão Oswaldo Cruz, Treze de Maio Street 1815, 01323-903, São Paulo, Brazil; 8 Department of Surgery, Duke University Health System, 2301 Erwin Road, Durham, 27705, USA

## Abstract

**Background:**

Industry standards provide rigorous descriptions of required data presentation, with the aim of ensuring compatibility across different clinical studies. However despite their crucial importance, these standards are often not used as expected in the development of clinical research. The reasons for this lack of compliance could be related to the high cost and time-intensive nature of the process of data standards implementation. The objective of this study was to evaluate the value of the extra time and cost required for different levels of data standardisation and the likelihood of researchers to comply with these levels. Since we believe that the cost and time necessary for the implementation of data standards can change over time, System Dynamics (SD) analysis was used to investigate how these variables interact and influence the adoption of data standards by clinical researchers.

**Methods:**

Three levels of data standards implementation were defined through focus group discussion involving four clinical research investigators. Ten Brazilian and eighteen American investigators responded to an online questionnaire which presented possible standards implementation scenarios, with respondents asked to choose one of two options available in each scenario. A random effects ordered probit model was used to estimate the effect of cost and time on investigators' willingness to adhere to data standards. The SD model was used to demonstrate the relationship between degrees of data standardisation and subsequent variation in cost and time required to start the associated study.

**Results:**

A preference for low cost and rapid implementation times was observed, with investigators more likely to incur costs than to accept a time delay in project start-up. SD analysis indicated that although initially extra time and cost are necessary for clinical study standardisation, there is a decrease in both over time.

**Conclusions:**

Future studies should explore ways of creating mechanisms which decrease the time and cost associated with standardisation processes. In addition, the fact that the costs and time necessary for data standards implementation decrease with time should be made known to the wider research community. Policy makers should attempt to match their data standardisation policies better with the expectations of researchers.

## Background

The adoption of information technology by the medical community is increasing, with patient records that used to be stored in different locations and in both paper and electronic formats, now gathered together as Electronic Medical Records (EMR)[1]. Besides the potential benefits for patients and providers, such as fewer medical errors and improved quality of care[2], there are also benefits for health care researchers, such as improving the understanding of clinical practice and outcome assessment[1]. However certain obstacles currently impair the achievement of these benefits. While a standardised system exists for coding diagnosis and procedure, medical terminology and clinical data such as labels used in laboratory tests and units of measurement are not always standardised and are therefore not easily accessible in discrete fields within the EMR[1].

In the academic research field there has also been an attempt to automate the clinical trial process and decrease the use of multi-part paper case report forms, leading to an increasing use of electronic data capture (EDC) tools. However, the rate of adoption has been relatively slow and such tools are being used in only ~30% of clinical studies, with many of those still retaining a paper back-up [3]. Furthermore, these tools still require improvement, since many are not yet ready to connect to or share data with other applications within the clinical trial process [4].

Millions of biomedical research datasets are generated every year, potentially yielding critical information which could significantly influence the way healthcare is practised. However this potential is often not realised, because different datasets typically use different 'term' definitions (definitions of variables in a database) [5], which prevents them from being integrated into larger datasets. Large integrated datasets are crucial because they have the statistical power necessary to confidently generalise findings from a sample to a wider population.

Data become much easier to handle if variables are referred to by the same term across different databases [6]. Data standards provide a rigorous description of data representation [7], allowing cooperation between researchers through the exchange of ideas and data [8]. Consistency in variable naming not only aids the integration of databases but also their analysis. This ensures compatibility across different clinical studies. The concept of standardised data includes the specification of data fields (variables) as well as value sets (codes) that encode data within these fields [9]. Despite their crucial importance, until now data standards have not been extensively used in clinical research [10].

Although the reasons for this lack of compliance are not clear, the cost and time-intensive nature of data standards implementation could be responsible. A study of primary care practices showed that despite the fact that many participants could see the motivation for and anticipated benefits related to data sharing, such as savings from improved coding, more efficient workflow for ancillary staff (e.g. laboratory results can be sent directly to a patient's practice EMR) and even the altruistic goal of improving public health, costs were identified as a significant barrier to health information exchange [11], and could be a factor related to the lack of compliance with data standards. However, in the industrial setting it has been demonstrated that data standards implementation, when applied in the start-up stage, can save not only money but also time in the long term [12].

In the initial phase of study, time and funding are required for the implementation of data standards. Despite the fact that this investment will pay for itself in the long term, it is feasible to believe that researchers would be less likely to make the effort to standardise their CRFs or EMRs. Nevertheless, the lack of quantification prevents adequate modelling of the minimum level of maturity required for widespread adherence among clinical researchers.

The economic aspects of using data standards from the perspective of bio-pharmaceutical companies, technology providers and contract research organisations have previously been studied[12]. To date, however, there has been no investigation of clinical researchers' willingness to spend the additional money and time needed for the implementation of these standards. Therefore, the objective of this study was to evaluate the value of the extra time and cost required for different levels of data standards maturity and the corresponding likelihood of researchers to comply with these standards.

## Methods

### Study sample

A list of ten investigators from the Hospital Alemão Oswaldo Cruz, Brazil and eighteen from Duke University Hospital, USA, was obtained from the administration department of each of these institutions. Professional clinical researchers who have taken part in at least one multi-site clinical trial participated in this study. Investigators were contacted by email and invited to respond to an online questionnaire offered through DADOS-Survey [13], a web application specifically designed for conducting surveys compliant with international survey guidelines [14]. Because the survey was anonymous, the project was exempt from informed consent, but nevertheless, approval from the Institutional Review Board was obtained from both participating institutions.

### Attributes and levels

The study began with a slide presentation on data standards implementation for all study participants. The presentation explained the advantages of data standards, such as the ability to merge data from the current study with other studies or administrative data, as well as the limitations of data standards, which included the increased cost and time necessary for project completion. Participants were presented with different examples of the cost and time necessary for study initiation using data standards, along with three possible data standards implementation levels, namely lite, intermediate and full (Table 1). A lite implementation level was defined as one involving low cost, a faster implementation time and a low level of standardisation. Intermediate implementation was defined as having mid-range cost and time for completion, as well as a greater level of standardisation. Full implementation was defined as one of high cost, a slower rate of completion and the highest level of standardisation.

**Table 1:** Possible data standards implementation levels - time and money parameters for defining the different levels of implementation.

Attributes	Levels
Additional cost of study	no additional cost, $10,000, $40,000 for US or R$5,000, R$20,000 for Brazil
Standards implementation	LITE, INTERMEDIATE, FULL
Additional time before initiation of study	no additional time, 1 month, 4 months

Since data standardisation can involve a variety of different steps, there is no common consensus regarding the explicit value of the 'average' amount of time and money spent on its implementation within different types of clinical research study. Therefore, we formed a focus group consisting of four clinical research investigators, who through a Delphi method [15] agreed time and dollar values that would be reasonable for the implementation of data standards in a medium-sized study. All investigators had experience of at least four previous clinical registries and experience of participating in at least one programme of data standardisation. After three rounds of the Delphi survey, the values (presented in Table 1) were agreed upon by all but one panel member (who disagreed on the amount of time required for the full protocol).

Twenty different data standardisation scenarios were identified for analysis (Table 2), after those that might generate contradiction were discounted. This arrangement resembled that of a conjoint analysis, but since only a small sample of researchers was available no modelling was performed. Instead, descriptive analysis was conducted so that the results could be fed into the SD model. Study participants were presented with these possible scenarios and were asked to choose one of two options presented in each scenario, which they would consider implementing in one of the clinical trials that they usually performed (constituting the average trial size and complexity for the group).

**Table 2:** The 20 possible scenarios of data standard implementation presented to the participants in the study.

	(A) Current		(B) Alternative
	Standards Implementation		Standards Implementation
choice 1	LITE	OR	INTERMEDIATE
choice 2	LITE	OR	INTERMEDIATE
choice 3	LITE	OR	INTERMEDIATE
choice 4	LITE	OR	FULL
choice 5	LITE	OR	FULL
choice 6	LITE	OR	FULL
choice 7	LITE	OR	FULL
choice 8	LITE	OR	INTERMEDIATE
choice 9	LITE	OR	INTERMEDIATE
choice 10	LITE	OR	INTERMEDIATE
choice 11	LITE	OR	INTERMEDIATE
choice 12	LITE	OR	FULL
choice 13	LITE	OR	FULL
choice 14	LITE	OR	FULL
choice 15	LITE	OR	INTERMEDIATE
choice 16	LITE	OR	INTERMEDIATE
choice 17	LITE	OR	INTERMEDIATE
choice 18	LITE	OR	FULL
choice 19	LITE	OR	FULL
choice 20	LITE	OR	FULL

Participants were asked to choose one from two possible data standard implementation levels.

*Time measure in months and money measure in American dollars for American researchers and in Brazilian reais for Brazilian researchers.

Once the participants had completed the surveys, data were extracted by the project coordinator and only questionnaires that were internally consistent were selected for statistical analysis.

### Statistical analysis

In order to estimate the effect of cost and time on investigators' willingness to adhere to data standards in their CRFs, a random effects ordered probit model was used [16], with change from current scenario taken as the dependent variable. In each model, dummy variables were created for the level of standards implementation (intermediate = 1, full = 2), additional cost of study (US$10,000, $40,000 for American researchers or R$5,000, R$10,000 for Brazilian researchers. Both currencies were included to allow for comparison between data collected in Brazil and the US) and additional time before initiation of study (one or four additional months).

### Modelling

**Figure 1:**
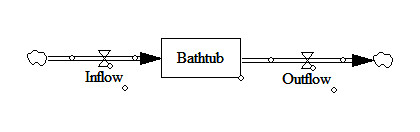
**Illustration of dynamic behaviour**.

Despite the fact that implementation time and cost stand to increase when data standards are used in a study, we believe that these variables can interact over time, leading to modification of the overall behaviour of a system - commonly referred to as dynamic behaviour. For example, inflow and outflow of water from a bathtub results in the generation of dynamic behaviour over a period of time (Figure1). System Dynamics (SD) analysis was used to investigate the behaviour of variables such as implementation time and cost over time. SD is essentially a set of tools that help the user understand and predict how systems (complex systems) behave over time[17]and is graphically represented by stocks (boxes), flows (thick arrows) and causal diagrams (thin arrows). A stock represents elements that can be measured and accumulated, and are regulated by the flows. A flow determines the rate of influx to, or efflux from the stock. Any other elements that influence the system are represented as a variable, with these relationships represented by causal diagrams. The SD model[18]was used to demonstrate the relationship between degrees of standardisation, cost of standardisation and time required to start the study. The model was created with the program Vensim PLE for Windows 5.9c[19].

Despite the fact that applying and using data standards in clinical studies can bring many advantages that could encourage researchers to adopt their implementation, there are also many associated drawbacks that could inhibit their use. From the survey, it was observed that an increase in cost and time to commence a study are unwanted aspects related to the use of data standards. Based on this information, we wanted to investigate how these variables, together with the increase in the number of standardised case report forms accumulated by a researcher, interacted and behaved over time, creating a situation that could change the researcher's perceptions regarding the implementation of data standards. Since SD is a set of tools used to help understand complex systems, we chose to use this strategy to help identify and explain the complex behaviour related to the use of data standards by unveiling the complexity behind its structure. We believe that by learning about the behaviour of this complex system, the advantages and drawbacks related the adoption of data standards can be identified and presented to researchers, so they can make an informed decision related to this subject.

## Results

### Probability of response

Probability of response results indicated a preference for free (definitely lite) standards implementation, with increasing probabilities of a choice of both intermediate and full standards with respect to their implementation at low, medium and high levels. This relationship, however, was only significant for full vis-à-vis lite implementation (p < 0.001), not vis-à-vis intermediate implementation (p = 0.228) (Table 3).

**Table 3:** Probabilities in relation to cost response.

	STANDARDS IMPLEMENTATION					
	INTERMEDIATE			FULL		
	LOW	MEDIUM	HIGH	LOW	MEDIUM	HIGH
**DEFINITELY LITE**	0.2690	0.2871	0.2999	0.1944	0.2728	0.3152
**PROBABLY LITE**	0.2980	0.2801	0.2579	0.2297	0.2319	0.1871
**DEFINITELY INTERMEDIATE**	0.0739	0.0641	0.0548			
**PROBABLY INTERMEDIATE**	0.1623	0.1341	0.1101			
**DEFINITELY FULL**				0.1318	0.1037	0.0679
**PROBABLY FULL**				0.3419	0.2048	0.000

The probability of response results with respect to time also show a preference for free alternatives (definitely lite), although this was not statistically significant compared to either intermediate or full standards (p = 0.116 and 0.496 respectively) (Table 4).

**Table 4:** Probabilities in relation to time response.

	PROPOSAL					
	INTERMEDIATE			FULL		
	FAST	MEDIUM	SLOW	FAST	MEDIUM	SLOW
**DEFINITELY LITE**	0.2738	0.2927	0.3048	0.2906	0.2850	0.2784
**PROBABLY LITE**	0.2929	0.2726	0.2476	0.1957	0.2010	0.2057
**DEFINITELY INTERMEDIATE**	0.0723	0.0618	0.0519			
**PROBABLY INTERMEDIATE**	0.1595	0.1294	0.1042			
**DEFINITELY FULL**				0.0829	0.0875	0.0921
**PROBABLY FULL**				0.1673	0.1809	0.1955

### Policy model

In our model we assume that the cost and time required to implement any given standard level decreases with the level of standards already accumulated. We describe here the behaviour of three variables (number of uniform datasets, implementation cost and implementation time) for a hypothetical period of five years. The degree of standardisation, cost of standardisation and time required to start the study were standardised on a scale from 1 to 10, with individual beta coefficients derived from the regression model using the normalised variables.

**Figure 2:**
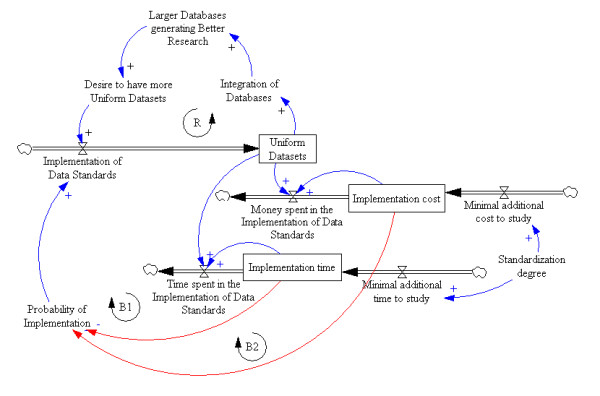
**System Dynamics Model**.

The general idea behind the model is that the implementation of data standards will lead to the generation of uniform datasets which integrate different databases, making it possible for the researcher to work with larger databases. This will result in higher quality research and publication, creating the desire for more uniform datasets which in turn will lead to the further implementation of data standards. This causal relationship between system variables is called a loop. In this case, the loop leads to the growth of the system, so is known as a reinforcing loop and is represented by the letter R in Figure 2.

If the system were composed of only a reinforcing loop, the growth of that system would be exponential. In a real-life scenario however, the cost and time required for standards implementation tend to act negatively, as demonstrated by our results, resulting in balancing loops B1 and B2. The first balancing loop (B1) represents the limitation caused by the additional time needed for data standardisation before study start-up. As a limitation it acts negatively, reducing the probability of implementation. Note that there is a causal relationship (represented by the arrows) between the number of uniform datasets and the time spent on implementation. This relationship will lead to lower levels of implementation over time in the function of the number of uniform datasets.

The second balancing loop (B2) represents the limitation caused by the extra costs of conducting a study involving standardised data. Here again there is a causal relationship between implementation cost and the number of uniform datasets, leading to lower costs over time depending on the number of datasets. In both balancing loops (B1 and B2), standardisation always requires some additional resource (time or money) to be consumed by the study.

**Figure 3:**
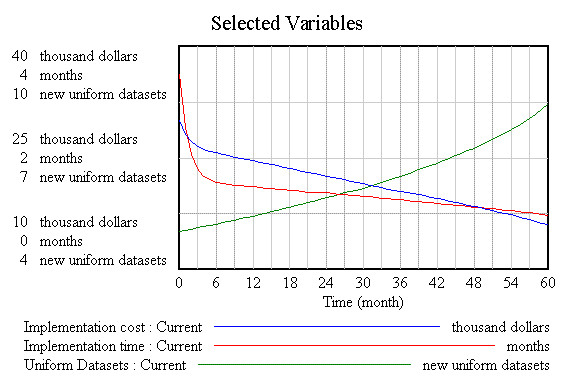
**Simulation**.

Figure 3 illustrates the behaviour of the variables 'number of uniform datasets', 'time' and 'cost' necessary for standardisation, analysed over time. As can be seen from this figure, the number of uniform datasets (green line) exhibits slow growth initially, but then increases slightly as a function of time and cost. The second variable, additional cost to the study (red line), decreases dramatically in the first six months because of the implementation of the initial datasets, and continues to drop throughout the period of analysis. The same pattern is exhibited by the remaining variable of time required for standardisation (blue line).

## Discussion

In the present study, whenever possible a researcher would prefer to implement the lowest possible level of standards that would make them minimally compliant. They also indicate that their preference is for a free alternative, rather than a more expensive option. This reflects the fact that the researchers perceive the increased expenditure and time needed to implement data standards as barriers to a study. Considering that many research projects rely on limited funding, this response could be expected. One interesting result, however, was that researchers prefer to pay rather than to delay the beginning of a project. Therefore it is not surprising that the implementation of data standardisation may be rejected if perceived as having the potential to delay project start-up.

Given the growth of clinical research and the corresponding increase in the volume of data produced, standards that facilitate data sharing, transformation and reuse are critical for maximising the amount of knowledge that can be gained [9], as well as helping to develop priceless repositories of knowledge [20]. In the industrial setting, the implementation of standards at the case report form stage of clinical trial development enhances data quality and facilitates communication between team members or partners [3,21]. The most widely recognised advantages of industrial data standardisation are cost and time savings. For example, a standard implemented at the beginning of a study provides greater returns on an investment and can result in resource savings of 60% [22] for a single clinical research study and 80% at the start-up stage [12]. Industry-wide standards are also efficient and effective in assessing the safety of new therapies [22]. According to the Institute of Medicine, the use of standards has helped to reduce expenditure in healthcare administration by 20 to 30% and has also yielded a cost reduction of 35% in the pharmaceutical industry [23].

Implementation of standards has also proven to have time-saving benefits with respect to the execution of research. Data organised in different databases and stored in different formats are difficult to gather and can thus delay research activities [24], with the use of standards helping shorten the time needed to complete clinical trials by as much as one year [25]. Sponsors have also been motivated to adopt standards with the aim of realising considerable time savings in the research process [20]. When data standards are used there is a definite reduction in the time required to create CRF and the database, perform audit checks, clean data, programme tables, lock the database after last subject visit, train new employees and conduct regulatory reviews [26].

Clinical Data Acquisition Standards Harmonisation (CDASH) is aimed at standardising the structure of a study's data and meta-data, promising to significantly expedite clinical studies as well as the exchange of data between sponsors and other participants in the process. An analysis by Gartner Inc. [27] indicated that when standards are implemented in the CRF development stage of a clinical study, significant time and cost savings may be achieved [21]. According to a collaboration between the Clinical Data Interchange Standards Consortium (CDISC) and the Health Information Management Systems Society (HIMSS), the process of enabling data to be entered only once in satisfying both the patient healthcare record and clinical research protocol requirements will save money and time, as well as enhancing data quality [4].

All the studies cited above indicate savings of time and cost obtained in an industrial setting. To the best of our knowledge, estimates of the amount of time and/or money necessary for the implementation of data standardisation in an academic environment have not been available until now. Since this sort of research tends to be characterised by reduced budgets and personnel compared with that developed by large pharmaceutical companies, such information must be provided for researchers so they can evaluate the adoption of standards specific to their research context. In the present study, the time and cost necessary for implementing data standardisation were analysed from the perspective of academic research. This is in contrast with investigations carried out by CDISC, who looked at the problem from an industrial perspective. In this context standardisation is often implemented upfront before the study is carried out, with the authors predicting cost savings as a result[3,12]. The objective of the survey performed in this study was to provide clinical researchers with parameters of the cost and time necessary to standardise a clinical trial in its initial phase of study.

To our knowledge, SD analysis has not been used to analyse the use of data standards by clinical researchers until now. SD analysis of the variables 'number of uniform datasets', 'implementation cost' and 'implementation time' indicated that in the initial phase of clinical study, both the extra time and cost necessary to implement data standardisation can act as a deterrent to their implementation. However over time, the number of uniform datasets accumulated by the researcher increases, leading to a decrease in the cost and time necessary for standardisation. It should be noted that this information was not available to the researchers at the moment they were asked to choose between levels of standardisation, although they were informed of the general advantages of using standardised data. This may represent a limitation of the present study, since it is possible that researchers would have made a different selection regarding the level of standardisation if they had been presented with this information and as such should be considered in future studies. Another limitation of the present study is that the small sample size precluded further analysis.

Given the difficulty of quantifying most of the elements involved in this complex system, a very simple model was created here in order to better understand the relationship between the elements of cost and time necessary for the implementation of data standardisation. As such, it should be pointed out that the SD model and simulations are not intended to act as a forecast, with the simulations created for hypothetical scenarios. While the developed model may be useful for explaining why standardisation is important, further studies are necessary in order to better understand this problem.

In this study, values of the time and money needed to implement data standards were based on a consensus amongst researchers; nevertheless, variations could occur. The amount of money defining each level (lite, intermediate or low) was defined in different currencies (American dollars for American researchers and Brazilian reais for Brazilian researchers) and only numerical values were used to determine levels of standardisation (lite, intermediate, full). In the statistical analysis, different currencies were not considered but rather only the levels of standardisation, so this should not have influenced the results.

The focus of the present work was to investigate the cost of and willingness for data standards implementation within a clinical trial design study, in which the researcher does not have the prior intention of using their data for research collaboration. In this respect, it is important to consider whether the costs associated with study standardisation will differ when the process is carried out prior to or after the completion of a study. Also, the motivation to standardise a study of a researcher who foresees collaboration and data sharing with another group, is definitely different from that observed in our results, since this framework was not considered in the survey. Lastly, our results are probably not suitable for generalised application to other aspects of clinical research design, for example when the implementation of standards is a required component of the submission of research data associated with a particular publication.

Before choosing between these levels of standardisation, researchers were informed of the advantages of using data standards (i.e. sharing and reuse of data) in their study. It should be borne in mind that owing to the competitive nature of the scientific research environment and the historical culture of not sharing data, this is not common practice amongst researchers [28]. In addition, many researchers were and are afraid that their findings could be stolen or misused when data are shared [29].

Despite the fact that the investigation of this psychological factor was not one of the initial objectives of this study, we believe that in order to expand the use of data standards within the scientific community, such influences must be considered and addressed. Lastly, the advantages of research standardisation must be emphasised, most importantly the fact that despite the initial investment required, researchers will actually save time and money as they accumulate a greater number of standardised studies. This must be made clear throughout the community if researchers intend to take full advantage of research data.

## Conclusion

In light of the above results, we believe that the identification of time and cost factors will allow for customisation of different approaches depending on the researcher's priorities. It will also allow research policy organisations to match their data standardisation policies more accurately to the expectations of researchers. Since the increased time and cost of starting a study were important factors influencing researchers not to use data standards, future studies should explore ways of creating mechanisms which decrease the time and cost associated with standardisation processes, thus facilitating their implementation. Other mechanisms should be created which increase the personal benefits for individual researchers using standards; for example, the increased likelihood of publication for studies in which data standards were implemented or where data sharing between multiple research groups occurred. Meta-data sharing should also be encouraged, since this should enhance data reuse and therefore indirectly encourage standardisation, which should in turn lead to the production of critical information benefiting healthcare research.

## Competing interests

The authors declare that they have no competing interests.

## Authors' contributions

LC wrote parts of the manuscript and reviewed it for intellectual content; GZ created the SD model, wrote parts of the manuscript and reviewed it for intellectual content; AZ wrote parts of the manuscript and reviewed it for intellectual content; JS wrote parts of the manuscript and reviewed it for intellectual content; EC participated in the development of study design and reviewed the manuscript for intellectual content; MN participated in the development of study design and reviewed the manuscript for intellectual content; GK was responsible for data collection and wrote parts of the manuscript; RP conceived the study, participated in its design and coordination and helped to draft the manuscript; All authors read and approved the final manuscript.

## References

[B1] Dean BB Use of Electronic Medical Records for Health Outcomes Research A Literature Review Medical Care Research and Review 2009 66 6 611 638 10.1177/1077558709332440 19279318

[B2] Hanna KE Research: Using electronic medical records to bridge patient care and research (White Paper) 2005 FasterCures, The Center for Accelerating Medical Solutions: Washington, DC

[B3] Kush R A Focus on Clinical Research at the Investigative Site 2007

[B4] Kush R Standard Bearers Envisage 2007 22

[B5] Karp PD A strategy for database interoperation Journal of Computational Biology 1995 2 4 573 86 10.1089/cmb.1995.2.573 8634909

[B6] Bodenreider O Stevens R Bio-ontologies: current trends and future directions Brief Bioinform 2006 7 3 256 74 10.1093/bib/bbl027 PMC1847325 16899495

[B7] Chalmers RJG Health care terminology for the electronic era Mayo Clinic proceedings Mayo Clinic 2006 81 6 729 31 10.4065/81.6.729 16770971

[B8] Lee ES Incorporating collaboratory concepts into informatics in support of translational interdisciplinary biomedical research International Journal of Medical Informatics 2009 78 1 10 21 10.1016/j.ijmedinf.2008.06.011 PMC2606933 18706852

[B9] Richesson RL Krischer J Data standards in clinical research: gaps, overlaps, challenges and future directions J Am Med Inform Assoc 2007 14 6 687 96 10.1197/jamia.M2470 PMC2213488 17712081

[B10] Kush RD Electronic health records, medical research, and the Tower of Babel N Engl J Med 2008 358 16 1738 40 10.1056/NEJMsb0800209 18420507

[B11] Fontaine P Health information exchange: participation by Minnesota primary care practices Archives of Internal Medicine 2010 170 7 622 9 10.1001/archinternmed.2010.54 20386006

[B12] Rozwell C Kush R Helton E Saving Time and Money Applied Clinical Trials 2007 16 6

[B13] Shah A DADOS-Survey: an open-source application for CHERRIES-compliant Web surveys BMC Med Inform Decis Mak 2006 6 34 10.1186/1472-6947-6-34 PMC1586000 16978409

[B14] Eysenbach G Improving the quality of web surveys: The checklist for reporting results of Internet e-surveys (CHERRIES) Journal of Medical Internet Research 2004 6 3 12 16 10.2196/jmir.6.3.e34 PMC1550605 15471760

[B15] Linstone HA Turoff M The Delphi method: techniques and applications 1975 xx Reading, Mass.: Addison-Wesley Pub. Co., Advanced Book Program 620

[B16] Agresti A An introduction to categorical data analysis Wiley series in probability and mathematical statistics 2007 xvii 2 Hoboken, NJ: Wiley-Interscience 372

[B17] System Dynamics Society 2009 http://www.systemdynamics.org/what_is_system_dynamics.html

[B18] Sterman J Business dynamics: systems thinking and modeling for a complex world 2000 xxvi Boston; Toronto: Irwin/McGraw-Hill 982

[B19] Vensim from Ventana Systems 2010 http://www.vensim.com/

[B20] Bleicher P Kubick W Kush R Special section on clinical research standards introduction Drug Information Journal 2007 41 3 369 371

[B21] Adams T Standardized CRF Data Elements - An Idea Whose Time has Come ACRP Monitor 2001 2

[B22] Kush R Healthcare and clinical research: a critical link through standards Community oncology 2007 4

[B23] Corrigan J Fostering rapid advances in health care learning from system demonstrations 2003 xiv The National Academies Press: Washington, D.C 94 25057641

[B24] Kush R Implementing Single Source: the STARBRITE proof-of-concept study J Am Med Inform Assoc 2007 14 5 662 73 10.1197/jamia.M2157 PMC1975790 17600107

[B25] McCourt B Data standards: At the intersection of sites, clinical research networks, and standards development initiatives Drug Information Journal 2007 41 3 393 404

[B26] Rozwell C Kush R Helton E CDISC Standards: Enabling Reuse Without Rework Applied clinical trials online 2006 http://appliedclinicaltrialsonline.findpharma.com/appliedclinicaltrials/CRO/Sponsor/CDISC-Standards-Enabling-Reuse-Without-Rework/ArticleStandard/Article/detail/334571

[B27] Gartner 2010 http://www.gartner.com

[B28] Campbell EG Data withholding in academic medicine: characteristics of faculty denied access to research results and biomaterials Research Policy 2000 29 2 303 312

[B29] Birnholtz J Bietz M Data at work: supporting sharing in science and engineering International ACM SIGGROUP conference on Supporting group work 2003 Sanibel Island, Florida, USA

[B30] Shah J Shah A Pietrobon R Scientific writing of novice researchers: what difficulties and encouragements do they encounter? Academic Medicine 2009 84 4 511 6 10.1097/ACM.0b013e31819a8c3c PMC6035752 19318791

[B31] Pietrobon R A suite of web applications to streamline the interdisciplinary collaboration in secondary data analyses BMC Med Res Methodol 2004 4 1 29 10.1186/1471-2288-4-29 PMC544191 15596017

[B32] Research on Research 2010 http://www.researchonresearch.org

